# Develop and Psychometric Testing an Instrument to Evaluate the Management of Digital Competence Sharing in Healthcare

**DOI:** 10.1155/jonm/9906301

**Published:** 2025-07-03

**Authors:** Mira Hammarén, Tarja Pölkki, Outi Kanste

**Affiliations:** ^1^Research Unit of Health Sciences and Technology, Faculty of Medicine, University of Oulu, Oulu, Finland; ^2^Medical Research Center Oulu, University Hospital, University of Oulu, Oulu, Finland

**Keywords:** digital competence, healthcare, instrument development, knowledge sharing, management, nursing, psychometric testing

## Abstract

**Aim:** To develop and psychometrically test an instrument to evaluate the management of digital competence sharing (MDCS) in healthcare.

**Background:** The rise of digital systems requires healthcare professionals to be digitally competent. Managers are responsible for ensuring that professionals possess the requisite digital competence and support their ongoing development.

**Methods:** This methodological study followed COSMIN guidelines for instrument development and involved three phases: (1) conceptualisation and item generation based on a qualitative framework; (2) face and content validity testing; and (3) structural validity and internal consistency evaluation. Content validity was assessed by an expert panel (*n* = 8) using the content validity index (CVI), and face validity was examined via pretesting with healthcare professionals (*n* = 8). Exploratory factor analysis (EFA) was conducted with a cross-sectional sample of healthcare professionals (*n* = 227) to determine structural validity. Cronbach's alpha was used to evaluate internal consistency.

**Results:** Seventy-five items were initially generated. The CVI exceeded the acceptable threshold of 0.90. Following the expert panel and pretesting, 40 items were retained for EFA. The final instrument, the MDCS, included 34 items across five factors: (1) creating a friendly and safe digital organisational atmosphere, (2) creating methods and practices of digital competence sharing, (3) identifying and utilising professionals' digital competence, (4) providing resources and opportunities for digital competence sharing, and (5) promoting digital competence sharing through leadership. Cronbach's alpha values for the factors ranged from 0.91 to 0.95.

**Conclusion:** The MDCS instrument demonstrates high construct validity and internal consistency, supporting its validity and reliability for assessing the MDCS in healthcare.

**Implications for Nursing Management:** This instrument can support nurse managers in identifying and enhancing digital competence sharing within their teams. Future studies should employ confirmatory factor analysis (CFA) to validate the MDCS structure across subgroups.

## 1. Introduction

After the rapid digital transformation, a gap has emerged between the development of digital healthcare and the digital competence of the workforce [1]. Digital competence involves confidently, critically, and responsibly using digital technologies for learning, work, and societal participation, combining knowledge, skills, and attitudes [2], and it intertwines with personal competencies such as social and cognitive skills, as well as ethical considerations [3]. In healthcare settings, digital competence is essential for routine tasks such as documenting patient information in electronic health records, using clinical decision support systems, managing digital appointments [4, 5], coordinating care through secure messaging, and employing digital tools for patient education and telehealth services [4]. Furthermore, ethical competence [5, 6] and attitudes towards information technology in patient care are recognised as essential to digital competence among healthcare professionals [5]. In this study, digital competence encompasses healthcare professionals' knowledge, skills, and attitudes towards digital technology, including their ability to effectively and meaningfully integrate digital technology into their work. This definition is informed by the prior literature [7, 8] but tailored specifically for the healthcare context of this study.

The conceptual framework of this study is based on the integration of knowledge management and digital competence sharing in healthcare organisations. Knowledge management is versatile, encompassing subtopics such as information management, knowledge-based management, and competence management [9, 10]. Central to knowledge management is the idea of optimising how knowledge is created, stored, shared, and applied within an organisation to support strategic objectives [9]. Knowledge sharing is integral to the knowledge management process, involving sharing expertise, exchanging information, and providing feedback on tasks and procedures [11]. Knowledge sharing occurs formally and informally [12]. Digital competence sharing refers to the processes through which individuals within an organisation exchange knowledge, skills, and practices related to the use of digital technologies, encompassing both formal mechanisms (e.g., training sessions and workshops) and informal exchanges (e.g., peer support and mentoring) [13].

Management plays a crucial role in supporting the digital competence of healthcare professionals [14–16]. The management of digital competence sharing (MDCS) refers to managerial actions that facilitate and enhance digital competence sharing. This includes providing resources and opportunities, creating methods and practices, managing digital competence, implementing intergenerational learning, creating a friendly and safe digital organisational atmosphere, and promoting digital competence sharing through leadership [13]. Effective digital competence management requires proactive leadership, strong interpersonal communication, and a commitment to continuous training and knowledge sharing [17]. Previous studies have found that management support, time resources [16, 18], and a positive workplace environment are key enablers of knowledge sharing [19]. Managers can strengthen these processes by promoting adaptability, enhancing resource use, and fostering respectful collaboration among professionals [20]. Collaborative learning embedded in daily work further supports professional development and strengthens team relationships [14]. By assessing and recognising the digital competence strengths of healthcare professionals, managers can leverage these competencies to benefit the work community [15]. However, challenges such as limited time and lack of formal training persist [21], highlighting the need for structured opportunities such as workshops and mentoring [14]. Furthermore, there are different learning abilities and needs for support in digital competence among professionals [22], emphasising the importance of management providing individual support and creating an open, supportive team culture [21].

Effective digital competence sharing and its management enhance overall digital competence and can accelerate the adoption of new technologies [8, 23]. Keeping abreast of digital competence is essential for providing effective patient care [18, 24], career longevity, and job satisfaction [25]. Conversely, insufficient digital competence can lead to inefficient workflows and higher turnover rates [22]. Despite the growing relevance of digital competence, healthcare professionals have expressed technology-related stress [22] and concerns regarding the harmful effects of technology on their work and well-being [26]. In particular, nurses have felt that the training and support related to technology are insufficient [18, 23]. In addition, intergenerational differences have been observed in digital competence among healthcare professionals; younger professionals are usually more able to accept and adapt to new technology [26], while technology-related stress has been identified particularly among older professionals [22].

To enhance digital competence in healthcare, assessment tools are needed to support the design and evaluation of initiatives such as organisation-wide digital competence programmes, interdisciplinary training, and embedded professional development [1]. Measuring the MDCS can help ensure that these strategies are appropriately implemented and effective. A validated instrument can help identify gaps in managerial support, guide resource allocation, and promote management practices in digital transformation. Previous studies have focused on developing instruments to measure knowledge-sharing behaviour [27] and the management perspective to measure knowledge management [28], but no instrument addresses digital competence sharing from the managerial perspective. In the healthcare context, instruments have been developed to evaluate knowledge management [29] and the knowledge management competencies of healthcare managers [10]. Regarding digital competence, instruments have been created to assess the digital competence of healthcare [6] and the informatics competency of managers [30]. While these instruments include aspects of managing digital competence, there is a lack of a comprehensive understanding of supporting and managing digital competence sharing, which highlights a research gap in healthcare. The instrument developed in this study aims to address these challenges by offering a theoretically grounded and validated instrument to evaluate how managers foster and facilitate digital competence sharing in healthcare organisations.

## 2. Methodology

### 2.1. Design

**Figure 1:**
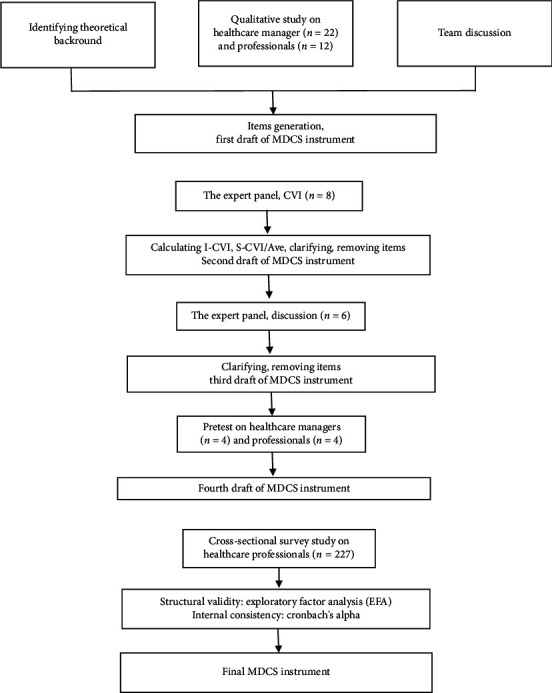
Phases of MDCS instrument development and psychometric testing. (a) Phase 1 conceptualisation and item generation (May 2021–November 2023). (b) Phase 2 face and content validity testing (January–May 2024). (c) Phase 3 structural validity and internal consistency testing (August–October 2024).

The development and psychometric testing of the MDCS instrument comprised three phases: (1) conceptualisation and item generation, (2) face and content validity testing, and (3) structural validity and internal consistency testing [31] (Figure 1). The instrument was developed in the Finnish language. The COSMIN checklist (COnsensus-based Standards for the selection of health status Measurement INstruments) was used to guide reporting and evaluate the methodological quality of studies on measurement properties [32] (Supporting 1).

#### 2.1.1. Phase I

The MDCS was conceptualised for this study, and an item pool was developed based on a theoretical framework [33] constructed from our earlier qualitative study [13]. This framework was derived from interviews with 22 healthcare managers and 12 healthcare professionals, who provided insights into their perceptions of the MDCS. Participating managers and professionals had practical experience with digital technologies due to the widespread use of digital health services and technological solutions in Finnish healthcare organisations. These include digital care pathways, virtual hospital systems, remote clinics, robotics, and other digital innovations. The qualitative data were analysed using inductive content analysis, and the results have been published [13]. The previous literature on knowledge sharing was reviewed [11, 12, 19] to support the findings of the qualitative study. The discussions of the research team supported the conceptualisation of the phenomenon and the creation of the item pool.

#### 2.1.2. Phase II

The content validity of the MDCS instrument was evaluated using the content validity index (CVI) method, with an expert panel (*n* = 8) assessing the clarity and relevance of the instrument's items. The item-level content validity index (I-CVI) and the scale-level content validity index (S-CVI) were calculated based on the experts' evaluations. The acceptable cut-off value for the CVI was established at 0.90 [34].

Although the target users of the instrument are healthcare professionals, the expert panel included researchers (*n* = 2), healthcare professionals (*n* = 2), and healthcare managers (*n* = 4), all of whom possessed expertise in digital healthcare and knowledge management. The inclusion of healthcare managers aimed to ensure that the instrument also reflects the broader organisational context in which digital competence is cultivated and shared. Purposive sampling was used as the sampling method [33]. The expert panel process was conducted in two rounds. In the first round, the experts (*n* = 8) completed a form to evaluate the clarity and relevance of the instrument's items using a four-point scale: (a) not relevant/clear, (b) needs some revision, (c) relevant/clear but needs minor revision, and (d) very relevant/clear. The instrument was modified based on feedback from the expert panel.

In the second round, six of the experts and the researcher (MH) convened to discuss the changes to the instrument and work towards a consensus. Finally, the experts had the opportunity to review and comment on the instrument. Following the expert panel, the face validity of the MDCS instrument was tested through a pretest with a new target group of four healthcare professionals and four managers. The pretesting aimed to determine the response time, assess the instrument's usability, and ensure that the target group understood the items.

A preliminary version of the MDCS instrument was developed, consisting of 40 items. A five-point Likert scale was used for responses (1-completely disagree, 2-partially disagree, 3-partially agree, and 4-completely agree), along with a “cannot say” option. The inclusion of the “cannot say” option was intended to accommodate situations where respondents may not have had sufficient information or experience to provide a meaningful answer. This option ensured greater accuracy by preventing forced responses that might not reflect actual perceptions or experiences [35].

#### 2.1.3. Phase III

##### 2.1.3.1. Item Analysis

An item analysis was conducted to evaluate the quality and performance of the preliminary items in the MDCS instrument. The analysis included the mean and standard deviation (SD) of items, examination of missing data, floor and ceiling effects, and corrected item-total correlations [36, 37]. Analyses were performed using IBM SPSS Statistics for Windows, version 29.0.

The data collected during the cross-sectional study were examined with regard to the “cannot say” response option on item levels. Since the “cannot say” option could not be assigned a specific value, it was coded as missing data. Items with more than 25% of “cannot say” responses were excluded from further analysis. The distribution of item scores was reviewed to assess potential floor or ceiling effects. An item was considered to show a floor or ceiling effect if more than 15% of respondents selected the highest or lowest possible score [36]. Corrected item-total correlations were calculated to examine the contribution of each item to the subscale. Items with low item-total correlations (< 0.30) were considered for revision or removal.

The structural validity of the instrument was assessed using exploratory factor analysis (EFA) with principal components factoring and Varimax rotation, which identified correlations among the variables and determined which items needed to be removed or regrouped [38]. The Kolmogorov–Smirnov test was conducted to indicate whether the data followed a normal distribution. The suitability of the data for EFA was assessed using the Kaiser–Meyer–Olkin (KMO) measure and Bartlett's test of sphericity. A KMO value greater than 0.60 was considered sufficient, and Bartlett's test was deemed significant at a *p* value of 0.05. Additionally, eigenvalues were utilised to evaluate the factors' ability to explain the variance in the variables. Factors with an eigenvalue greater than one were included in the factor model. Factor loadings less than 0.400 were excluded, enhancing accuracy [39].

The instrument's internal consistency was assessed using Cronbach's alpha [39]. Cronbach's alpha values were calculated for each factor and the entire instrument. Alpha values exceeding 0.70 were considered acceptable [40].

### 2.2. Participants

The participants in phases III and IV were healthcare professionals (*N* = 4500, *n* = 227) from three public and one private healthcare organisation in Finland. Public healthcare organisations provided health and social services, encompassing primary and specialised medical care, to multiple municipalities. The private healthcare organisation covered a hospital that delivered specialised medical care services. The inclusion criteria for professionals were licensed healthcare professionals (e.g., nurses, midwives, practical nurses and physiotherapists). The participants were selected using convenience sampling [33]. Units providing elderly healthcare services, primary and specialised healthcare services (including outpatient and ward services), and rehabilitation services were selected from each organisation to ensure diverse participation from healthcare professionals. These units were chosen due to their extensive and diverse healthcare professional. The sample size was determined by the need for at least five participants per item to ensure the structural validity of the instrument and to assess its internal consistency [39].

### 2.3. Data Collection

Data for phase III were collected through an electronic survey using a cross-sectional study design conducted via Webropol from August to October 2024. The survey invitations were distributed to healthcare professionals by organisational contact persons, who forwarded the electronic survey to the participants' email addresses. Participants received reminders to complete the survey twice every 2 weeks. The survey included the preliminary version of the MDCS instrument described above and the background questions (Table 1).

A total of 248 healthcare professionals participated in the survey, yielding a response rate of 5.5%. Respondents who selected “cannot say” for more than half of the items were excluded from the analysis (*n* = 21). Consequently, the final dataset comprised 227 healthcare professionals. Most of the professionals were female, with a mean age of 45. Registered nurses constituted 57% of the respondents. Furthermore, over 50% of the respondents were employed in public specialised healthcare.

### 2.4. Ethical Considerations

The study followed principles and practices of scientific research, including honesty, general diligence and accuracy [41]. Research permits were obtained from target organisations following their policies. Finnish legislation did not require ethical committee approval, as the study did not involve patients, minors, or interventions affecting participants' physical or mental integrity [42, 43]. The survey invitations included information about the study's aims, methods, ethical considerations, and data protection. Participants received written information about the voluntary participation and the option to withdraw at any time. They had the opportunity to discuss the study and ask questions with the researchers. Written informed consent was obtained from all participants. Informed consent was provided electronically at the beginning of the Webropol survey. The study collected only the personal data necessary for its purposes. The data were stored on a secure network disk, accessible only through separate user IDs, and will be discarded after the results are published [44].

## 3. Results

The results are presented according to the three phases of instrument development: (1) conceptualisation and item generation, (2) face and content validity testing, and (3) structural validity and internal consistency testing.

### 3.1. Phase I Conceptualisation and Item Generation

A framework was developed for this study based on a prior qualitative study. Six categories were identified, which were as follows: (1) providing resources and opportunities for digital competence sharing, (2) creating methods and practices for digital competence sharing, (3) managing healthcare professionals' digital competence, (4) implementing intergenerational learning, (5) creating a friendly and safe digital organisational atmosphere, and (6) promoting digital competence sharing through leadership. Based on these categories, a total of 75 items were developed.

### 3.2. Phase II Face and Content Validity Testing

The I-CVI and S-CVI were calculated in the first round of an expert panel. The relevance at the item level ranged from 0.63 to 1, and the clarity varied from 0.63 to 1. S-CVI/Ave had a value of 0.93 for relevance and 0.95 for clarity, indicating high content validity. Based on the expert panel's evaluation and written feedback, items with an I-CVI value below one were modified or removed, while those below 0.75 were removed. In addition, a few items that received one were also removed based on the experts' feedback. As a result, 19 items were clarified, and 32 items were removed. Furthermore, one item was included in the instrument based on expert feedback. After the first round, 44 items remained in the instrument, with the following six subscales presented in Phase I.

The instrument's structure was modified during collaborative discussions with experts in the second round. Items in the ‘Implementation of Intergenerational Learning' subscale were reallocated to managing digital competence and creating methods and practices for sharing digital competence as they aligned more appropriately with these areas. Based on the discussion, minor modifications, including adding examples, were made to enhance clarity. Four items were removed as they were identified as redundant and repetitive within the instrument. Finally, the experts received the completed instrument for review, and no changes were made at this stage. After the second round, 40 items remained in the instrument, with the five following subscales: (1) creating a friendly and safe digital organisational atmosphere, (2) creating methods and practices of digital competence sharing, (3) managing professionals' digital competence, (4) providing resources and opportunities for digital competence sharing, and (5) promoting digital competence sharing through leadership. More detailed information on the instrument's subscales and the number of items at each phase of its development is provided in the supporting information (Supporting Table 1).

Eight participants, including four healthcare managers and four professionals, pretested the MDCS instrument in a pilot study. Based on their feedback, minor adjustments were made to two background questions, with no changes to the instrument items. The responses from this pretest were not included in the data from the cross-sectional study.

### 3.3. Phase III Structural Validity and Internal Consistency Testing

#### 3.3.1. Item Analysis

Five items were excluded from further analysis because they had more than 25% missing responses (“cannot say” option). Descriptive statistics for each item, including mean, SD, and the presence of floor or ceiling effects, are presented in Table 2. Several items demonstrated floor effects, and nine items demonstrated ceiling effects. The mean item scores ranged from 2.090 to 3.048, with SDs ranging from 0.778 to 1.024, indicating variability in how respondents rated different aspects measured by the instrument. The corrected item-total correlations for the remaining items ranged from 0.653 to 0.848, all exceeding the recommended threshold of 0.30.

#### 3.3.2. Structural Validity and Internal Consistency Testing

The KMO measure was 0.948, and Bartlett's test of sphericity was significant (*χ*^2^ = 4163.680, df = 595, *p* < 0.001), indicating that the sample size was sufficient for factor analysis. EFA was performed using principal component analysis with Varimax rotation, resulting in a five-factor model with 34 items. One item (item 34) loaded onto a new factor was reassigned to a more appropriate factor in terms of content. Additionally, two items (items 31 and 32) were reassigned to the new factor due to their content alignment.

In the MDCS instrument, the total variance explained by the five-factor model was 76.75%. The first factor, *Creating a friendly and safe digital organisational atmosphere*, explained 61.74% of the total variance. The second factor, *Creating methods and practices for digital competence sharing*, explained 4.79% of the total variance. The third factor, *Identifying and utilising professionals' digital competence*, explained 3.82% of the total variance. The fourth factor, *Providing resources and opportunities for digital competence sharing*, explained 3.45% of the total variance. The fifth factor, *Promoting digital competence sharing through leadership*, explained 2.97% of the total variance. The Cronbach's alpha coefficients for the MDCS instrument factors ranged from 0.91 to 0.95 (Table 2).

#### 3.3.3. The Scoring and Structure of the MDCS Instrument

The developed MDCS instrument consists of five subscales with a total of 34 items, rated on a five-point Likert scale for responses (1-completely disagree, 2-partially disagree, 3-partially agree, and 4-completely agree), along with a “cannot say” option (coded missing value). Higher scores indicate greater levels of the MDCS. The score ranges and descriptive statistics for the subscales are presented in Table 3.

## 4. Discussion

This study developed and psychometrically tested an instrument designed to evaluate the MDCS among healthcare professionals, considering five distinct areas from their perspective. The first factor pertains to cultivating a supportive and secure digital environment by valuing digital competence and effectively communicating the benefits of digitalisation. The second concerns the establishment of methods and practices that foster collaboration and knowledge sharing. The third emphasises the identification and utilisation of professionals' digital strengths alongside the encouragement of mentoring. The fourth highlights the provision of resources and conditions that facilitate the digital competence sharing. Finally, the fifth factor reflects leadership behaviours such as demonstrating digital proficiency, anticipating future needs, and addressing potential barriers. Healthcare managers can utilise this instrument to facilitate and enhance the digital competence of healthcare professionals. The MDCS instrument examines the MDCS comprehensively, considering aspects such as atmosphere, practices and methods, competence, resources, and leadership. Previous instruments have evaluated knowledge sharing and leadership in a more restricted area, such as examining the relationships between transformational leadership and the knowledge-sharing atmosphere and behaviour [11]. This instrument provides a deeper understanding of the management role by exploring the utilisation of existing digital competence within the work community and examining how management can facilitate sharing this competence.

In the first phase, the MDCS was initially defined based on a prior qualitative study, identifying six key categories related to leadership, resources, practices, competence management, intergenerational learning and organisational culture. This original conceptualisation informed the development of 75 items. After expert panel and psychometric analysis, the instrument was refined into five factors, resulting in a more focused final definition that emphasises a leadership-driven, supportive organisational atmosphere, practical methods and resource provision to promote digital competence sharing.

The content validity of the instrument was established through expert evaluation, ensuring that the items comprehensively represented the domain of MDCS. Face validity was further strengthened through pilot testing with a sample of healthcare professionals, who confirmed the relevance and clarity of the items [34]. Nevertheless, future studies could further enhance face validity by involving a broader range of professionals from diverse healthcare contexts. Item analysis revealed the presence of floor effects within the MDCS instrument. The floor effect indicates that the range of response variability may be restricted for certain items, particularly at the lower end of the scale [37].

Structural validity was examined using EFA, which supported the instrument's theoretical framework. The factor structure corresponded well to the predefined constructs, providing preliminary evidence of construct validity. In the MDCS instrument, the organisational atmosphere plays a vital role in digital competence sharing, and it describes the environment for knowledge sharing from the perspective of digital competence, emphasising a manager's duty to support a pressure-free and open environment where everyone can express their digital competence needs and everyone's expertise is valued. Similar elements have been explored in instruments evaluating the knowledge-sharing atmosphere [11]. The MDCS instrument assessed the allocation of dedicated time and opportunities for competence sharing, a factor similarly identified as crucial in knowledge management contexts [28]. Furthermore, the instrument highlights the importance of managers encouraging the sharing of digital competence by leading by an example and promoting the development of digital competence among professionals. The role of the frontline manager and top-management support was identified as an essential part of knowledge management and sharing in previous instruments [9, 10, 28].

However, hypothesis testing was not conducted in this study [36]. This instrument identified recognising and utilising generational differences and strengths, which have not been considered in previous knowledge management instruments [28, 29]. Previous studies have identified a negative association between age and digital competence [26]. Future studies should include hypothesis testing, such as examining the relationship between age and instrument scores, to further confirm the instrument's construct validity and to strengthen the evidence base for its use in different healthcare settings. The instrument demonstrated high internal consistency across factors, with Cronbach's alpha values exceeding 0.90. A Cronbach's alpha value of 0.70 or higher is generally considered acceptable, values above 0.80 are considered good, and values above 0.90 may indicate high internal consistency [40]. These findings suggest that the items within each construct reliably measure the same underlying concept. However, as with all self-report measures, the possibility of inflated reliability due to similar item wording cannot be entirely excluded.

The MDCS instrument differs from previous instruments related to knowledge management of knowledge sharing by explicitly addressing generational differences and strengths, which have not been considered before [28, 29]. However, similar to prior instruments, it emphasises managers' roles in evaluating competence and allocating time for competence sharing [28]. Leadership support in promoting digital competence aligns with the recognised roles of frontline managers and top management in knowledge management and sharing [10, 28, 29]. Additionally, the MDCS highlights formal and informal practices for facilitating professional interaction, a feature partly reflected in existing knowledge management instruments [29].

The MDCS instrument included five statements that over 25% of the professionals could not evaluate. These items relate to the manager's digital competence, current knowledge of digital advancements and development of these competencies. This suggests that professionals may need to be more aware of their manager's digital expertise. Furthermore, the professionals could not evaluate whether the managers consider different learners in digital competence or recognise the benefits of digital competence sharing. However, it is important to acknowledge that assessing managers' competence by their subordinates may introduce response bias, as professionals might feel hesitant to evaluate their managers. This potential reactivity should be taken into account when interpreting the results.

### 4.1. Limitations

The study had some limitations. The items in the instrument were developed based on empirical data. A systematic review would have enhanced the instrument's initial phase and theoretical framework by providing a more robust foundation. However, given the limited number of studies on the subject, a systematic review was not feasible. Another limitation concerns the psychometric properties assessed. While the study focused on content validity, internal consistency and construct validity through EFA, other important aspects—such as criterion validity, test-retest reliability, and responsiveness—were not examined, as recommended by the COSMIN guidelines. This study did not perform confirmatory factor analysis (CFA), which is essential for validating the factor structure. Future studies should include CFA to validate the MDCS instrument's structure in different subgroups. The low response rate (5.5%) may limit the study results. However, the sample size is deemed sufficient, as each instrument item received more than five responses [34]. A notable limitation is the demographic composition of the sample, with most of the participants being female and more than half being nurses. This reflects the gender and profession distribution in Finland's healthcare sector, where females and nurses constitute most of the workforce. One limitation was that a random sampling method was not used to select participants.

## 5. Conclusions

The MDCS instruments were demonstrated to be valid and reliable for assessing the MDCS from the perspective of healthcare professionals. The instrument is suitable for use in various healthcare settings. However, the MDCS instrument requires further studies to enhance its validity and applicability across more diverse target groups, such as physicians and social care professionals. CFA can be employed to enhance the structural validity of the MDCS instrument, allowing for the testing of the factor structure and providing a more rigorous assessment of the instrument's validity. Furthermore, this instrument should be developed and subjected to psychometric testing for application by healthcare managers. The findings can be leveraged to promote digital competence sharing and reduce disparities among professionals by developing and implementing supportive methods and operational strategies. Additionally, the MDCS instrument can be employed to enhance healthcare managers' competence and assess the effectiveness of training and education interventions to promote the ability to manage digital competence sharing.

### 5.1. Implications for Nursing Management

The MDCS instrument offers a novel and practical contribution to nursing management by providing a structured means to evaluate and support digital competence sharing in healthcare organisations. It addresses a critical gap in current research by focussing on individual digital competence and the managerial processes that enable effective knowledge sharing and digital competence development across teams.

For nurse managers, the instrument enables the identification of strengths and gaps in digital competence management, supporting the creation of targeted strategies to foster a culture of continuous learning and collaboration. By integrating the MDCS instrument into leadership development and training programs, nursing managers can enhance their ability to lead digital transformation efforts effectively. In nursing practice, the instrument provides a valuable tool for assessing and developing managers' competence in supporting digital competence sharing. Its application can inform professionals' development, guide policy and operational planning and contribute to more effective healthcare management.

## Figures and Tables

**Table 1:** Characteristics of the participants (*n* = 227).

Characteristics	*n*	%
Gender		
Female	202	89.0
Male	19	8.4
Prefer not to say	6	2.6
Profession		
Registered nurse	129	56.8
Practical nurse	43	18.9
Physical therapist	18	7.9
Midwife	6	2.6
Dental hygienist/nurse	6	2.6
Public health nurse	5	2.2
Speech therapist	4	1.8
Occupational therapist	3	1.3
Other^1^	13	5.7
Organisation		
Public specialised healthcare	135	59.5
Public primary healthcare	64	28.2
Private specialised healthcare	24	10.6
Private primary healthcare	4	1.8
Clinical working environment		
Inpatient^2^	117	51.5
Outpatient^3^	110	48.5
Educational level		
Master's degree or higher	33	14.5
Bachelor's degree	97	42.7
Second level degree or lower	97	42.7

	**Mean (SD)**	**Range in years**

Age	45.3 (11.6)	20–69
Work experience		
In current work	10.8 (9.6)	0–43
In healthcare	16.9 (10.7)	0–45

^1^Radiographer, rehabilitation counsellor, psychologist, audiologist, pharmacist, and nutritionist.

^2^Hospital ward, intensive care unit, operating theatre, delivery room, and nursing home.

^3^Outpatient clinics, day surgery unit, home care, assisted living, rehabilitation services, dental care, and laboratory.

**Table 2:** Exploratory factor analysis of the management of the digital competence sharing instrument.

Factors describing the management of digital competence sharing in healthcare	Mean SD	Factor loading				
		1	2	3	4	5
*Factor 1: Creating a friendly and safe digital organisational atmosphere*						
1. The manager respects of all ages and their digital competence	2.826^b^ 0.974	**0.734**	0.289	0.298	0.277	0.136
2. The manager requires professionals to develop their digital competence without excessive pressure	2.827^b^ 0.898	**0.714**	0.308	0.288	0.296	0.221
3. The manager values digital competence	3.022^b^ 0.914	**0.710**	0.229	0.395	0.168	0.192
4. The manager creates an atmosphere where professionals feel confident to express their digital competence needs	2.736^b^ 0.962	**0.666**	0.397	0.210	0.272	0.241
5. The manager gives every professional the opportunity to develop their digital competence	2.471^a^ 0.978	**0.663**	0.286	0.156	0.429	0.267
6. The manager creates an atmosphere that encourages the development of digital competence	2.597 0.941	**0.647**	0.437	0.205	0.300	0.246
7. The manager communicates the benefits related to digitalisation	2.509 0.902	**0.646**	0.099	0.195	0.209	0.426
8. The manager provides information about changes related to digitalisation	2.788^b^ 0.892	**0.640**	0.177	0.293	0.251	0.363

*Factor 2: Creating methods and practices for digital competence sharing*						
9. The manager encourages professionals to create guidelines for each other on the use of digital solutions	2.486^a^ 0.955	0.233	**0.797**	0.201	0.193	0.134
10. The manager encourages professionals to support each other in digital competence	2.792^b^ 0.870	0.261	**0.734**	0.354	0.233	0.118
11. The manager encourages professionals to practice using digital solutions together	2.607 0.887	0.277	**0.729**	0.279	0.243	0.194
12. The manager encourages professionals to share digital competence with each other, e.g., through joint discussions	2.664^b^ 0.924	0.323	**0.697**	0.310	0.305	0.207
13. The manager enables working conditions where professionals can work close to each other to share digital competence	2.414^a^ 0.928	0.207	**0.552**	0.062	0.259	0.524
14. The manager ensures that IT responsible persons are trained to share their digital competence	2.379^a^ 0.920	0.142	**0.536**	0.364	0.479	0.205
15. The manager promotes collaboration among professionals of different ages to advance sharing digital competence	2.175^a^ 0.882	0.299	**0.530**	0.418	0.363	0.298
16. The manager facilitates pair work to share digital competence	2.172^a^ 0.959	0.294	**0.469**	0.381	0.349	0.247

*Factor 3: Identifying and utilising professionals' digital competence*						
17. The manager recognises the strengths of different generations in digital competence	2.490^a^ 0.986	0.198	0.283	**0.765**	0.238	0.233
18. The manager recognises the strengths of professionals in digital competence	2.594^a^ 1.024	0.357	0.200	**0.751**	0.209	0.249
19. The manager assigns tasks according to the digital competence of professionals	2.270^a^ 0.963	0.279	0.277	**0.685**	0.351	0.202
20. The manager leverages the strengths of different generations to promote professionals' digital competence	2.360^a^ 0.935	0.247	0.489	**0.664**	0.228	0.149
21. The manager recognises the different needs of generations in developing digital competence	2.211^a^ 0.913	0.308	0.191	**0.641**	0.383	0.219
22. The manager identifies factors affecting professionals' digital competence	2.337^a^ 0.967	0.295	0.418	**0.638**	0.250	0.280
23. The manager guides professionals with strong digital competence to mentor others	2.550^a^ 0.962	0.237	0.535	**0.555**	0.200	0.249

*Factor 4: Providing resources and opportunities for digital competence sharing*						
24. The manager allocates time for digital competence sharing	2.090^a^ 0.776	0.287	0.214	0.252	**0.778**	0.030
25. The manager allocates time for learning new digital solutions	2.194^a^ 0.879	0.278	0.316	0.239	**0.704**	0.182
26. The manager organises working conditions that enable digital competence sharing alongside work	2.142^a^ 0.816	0.269	0.371	0.215	**0.688**	0.179
27. The manager considers the workload of task-related digital competence sharing (responsible user) equally	2.180^a^ 0.852	0.304	0.167	0.323	**0.666**	0.320
28. The manager provides opportunities for digital competence sharing (e.g., team meetings and joint gatherings)	2.347^a^ 0.952	0.299	0.227	0.439	**0.600**	0.244

*Factor 5: Promoting digital competence sharing through leadership*						
29. The manager has digital competence	3.048^b^ 0.909	0.310	0.101	0.342	0.075	**0.765**
30. The manager leads the digital competence sharing by an example	2.495 0.984	0.371	0.271	0.313	0.149	**0.675**
31. The manager ensures that professionals have the necessary digital competence	2.317^a^ 0.909	0.275	0.332	0.250	0.460	**0.543**
32. The manager anticipates the needs related to professionals' digital competence	2.230^a^ 0.868	0.405	0.211	0.363	0.307	**0.533**
33. The manager identifies factors that prevent or weaken professionals' digital competence sharing	2.531^ab^ 1.010	0.227	0.354	0.157	0.474	**0.531**
34. The manager takes care of the development of professionals' digital competence	2.330^a^ 0.902	0.335	0.335	0.232	0.475	**0.461**
Eigenvalue		20.990	1.628	1.298	1.171	1.008
Percentage of variance explained		61.736	4.788	3.819	3.446	2.965
Total proportion of variance explained by the factor model		76.754				
Cronbach's alpha		0.949	0.912	0.937	0.952	0.911
Cronbach's alpha on the total scale		0.981				

*Note:* Extraction method: principal component analysis with Varimax rotation. Bold values are included in the factor.

^a^Floor effect.

^b^Ceiling effect.

**Table 3:** Score ranges and descriptive statistics for the subscales of the management of the digital competence sharing instrument.

Subscales of the MDCS instrument	Score range	Observed score range	*n*	Mean SD
Creating a friendly and safe digital organisational atmosphere (8 items)	8–32	8–32	147	22.150 6.373

Creating methods and practices for digital competence sharing (8 items)	8–32	8–32	181	19.348 6.270

Identifying and utilising professionals' digital competence (7 items)	7–28	7–28	156	16.455 6.037

Providing resources and opportunities for digital competence sharing (5 items)	5–20	5–20	185	10.621 3.641

Leadership in promoting digital competence sharing (6 items)	6–24	6–24	154	15.195 4.667

## Data Availability

The data that support the findings of this study are available from the corresponding author upon reasonable request.

## Supporting Information

The COnsensus-based Standards for the selection of health status Measurement INstruments (COSMIN) checklist [32], which is used to guide reporting, is presented in the supporting information. Supporting Table 1: The subscales and items of the management of the digital competence sharing instrument.
