# Supplementary material for: Develop and Psychometric Testing an Instrument to Evaluate the Management of Digital Competence Sharing in Healthcare
Source: J Nurs Manag. 2025 Jul 3;2025:9906301. doi: 10.1155/jonm/9906301 (PMC12245504; doi:10.1155/jonm/9906301)
Supplement: Supporting Information — Additional supporting information can be found online in the Supporting Information section. [file 9906301.f1.zip › COSMIN checklist.docx]

**Supplementary material:** The COnsensus-based Standards for the selection of health status Measurement INstruments (COSMIN) guideline checklist.

Mokkink, L. B., Terwee, C. B., Knol, D. L., Stratford, P. W., Alonso, J., Patrick, D. L., Bouter, L. M., & de Vet, H. C. (2010). The COSMIN checklist for evaluating the methodological quality of studies on measurement properties: A clarification of its content. BMC Medical Research Methodology, 10(1), 22. https://doi.org/10.1186/1471-2288-10-22

| **Report section:** | **Item description** | **Page nro.** |
| --- | --- | --- |
| **Title** | | |
| The name of instrument | The name of the instrument is included in the title. | 1 |
| Measurement property | The psychometric testing is included in the title. | 1 |
| Study sample | The study sample it not included in the title. | - |
| **Abstract** | | |
| The name of instrument | The name of the instrument and the type of instrument is reported. | 1 |
| Measurement property | The validity and reliability of the instrument development is described. | 1 |
| Design | The study design is described. | 1 |
| Sample | The sample size is described. | 1 |
| Methods | Methods are described. The instrument development consisted of four phases: 1) conceptualization and item generation, 2) face and content validity testing, and 3) structural validity testing, and 4) internal consistency testing. | 1 |
| Result | The content validity index, exploratory factor analysis and Cronbach`s alpha values were described. | 1 |
| Discussion/ Conclusion | The instrument's usability was described. | 1 |
| **Introduction and background** | | |
| Name and describe the PROM of interest | The name of the instrument is not mentioned in introduction, the instrument is described in greater detail in methods. | - |
| Target population | The management of digital competence sharing among healthcare professionals is detailed | 2-4 |
| Citation of the original development of instrument | Citations are provided for the theoretical framework, which is based qualitative study. | 3 |
| State of knowledge and rationale | Previously designed instruments described. | 4 |
| Definitions | Definitions of all used terms are presented. | 2-4 |
| Objectives and Hypothesis | The objectives of the study were presented in methods, and no hypothesis was set for this study. | 4 |
| **Methods** | | |
| Study design | The study design and methods are described. | 5 |
| Participants | Phase 2: Expert panel (n = 8) and pilot study (n =8), Phase 3 and Phase 4: healthcare professionals (n=227). | 5-6 |
| Instrument administration | The data were collected between august to October 2024 using Webropol -software. | 7 |
| Data collection procedure | The data collection time frame and the number of reminders were described. | 7 |
| Power /sample size calculation | The sample size was estimated by counting 5 participants per instrument item. | 6 |
| Statistical analyses | Statical analyses are described in methods. | 6 |
| Missing data | Missing data are reported. | 9 |
| Post hoc analysis | Not applicable in this study | - |
| **Results** | | |
| Missing data | Missing data are reported. | 9 |
| Participants characteristics | The participant characteristics are reported in results and Table 1. | 9 (Table 1) |
| Sample size | The sample size for each background variable is described in Table 1. | (Table 1) |
| **Discussion** | | |
| MP evidence | Measurement properties are described in the results and in a Table 2. | 9 |
| Practical relevance | Instrument`s practical relevance is discussed in discussion and conclusion | 10-12 |
| Strength and limitations | The strengths and limitations of the study are described. | 12 |
| Generality | Generalizability is discussed in the limitation section. | 12 |
| Instrument changes | Further development of the instrument is discussed in the conclusions section. | 12-13 |
| Future research | Future research recommendation is discussed in the conclusion section. | 12-13 |
| **Conclusion** | | |
| Conclusion | The overall conclusion is that the MDCS instrument is a valid and reliable instrument. | 12-13 |
| **Other information** | | |
| Conflict of interest | Conflict of interest are discussed. | 13 |
| **Content Validity** | | |
| Relevace | An expert panel (n=8) evaluated the relevance of each item. | 8 |
| Comprehensiveness | An expert panel evaluated the clarity of each item and provide additional comment. | 8 |
| Comprehensibility | The pretest (n=8) was conducted to evaluate comprehensibility. | 8 |
| Relevance results | 32 items were removed based on the experts’ feedback. | 8 |
| Response options and recall period | The pretest (n=8) evaluated the suitability of the response options, the structure of the instrument, and the length of the response period. | 8 |
| Comprehensiveness results | 19 items were clarified based on the experts’ feedback. | 8 |
| Comprehensibility results | None of the items needed to be modified. | 8 |
| **Structural validity** | | |
| Factor analyses: classical test theory | An exploratory factor analysis (EFA) using Principal components analysis with Varimax rotation was conducted because no clear a priori hypothesis was available. The cut-off loading for EFA was set and the number of factors was determined based on the theoretical framework and by counting the number of eigenvalues. | 9 |
| Item Response theory (IRT) analyses | Not applicable in this study | - |
| **Internal consistency** | | |
| Unit of measurement | Cronbach’s alpha was used to assess internal consistency. | 10 (Table 2) |
| Continuous scores | Cronbach's alpha values were calculated for each of factors (Table 2). | (Table 2) |
| Dichotomous scores | Not applicable in this study | - |
| **Reliability** | | |
| PROM administrations | Cronbach's alpha was used to assess the internal consistency. | 10 |
| Statistical analyses | Cronbach's alpha was used to assess the internal consistency. | 10 |
| Methods to improve reliability | Cronbach's alpha was used to assess the internal consistency. | 10 |
| **Criterion validity** | | |
| Criterion | It was not done in this study, because there were no instruments to be used as criterion. | - |
| Continuous scores | It was not done in this study, because there were no instruments to be used as criterion. | - |
| Categorical scores | It was not done in this study, because there were no instruments to be used as criterion. | - |
