# Supplementary material for: Develop and Psychometric Testing an Instrument to Evaluate the Management of Digital Competence Sharing in Healthcare
Source: J Nurs Manag. 2025 Jul 3;2025:9906301. doi: 10.1155/jonm/9906301 (PMC12245504; doi:10.1155/jonm/9906301)
Supplement: Supporting Information — Additional supporting information can be found online in the Supporting Information section. [file 9906301.f1.zip › Supplementary Table 1.docx]

Supplementary Table 1. The subscales and items of the Management of digital competence sharing instrument

| Phase I Conceptualisation and item generation | |
| --- | --- |
| May 2021-November 2023  Conceptual framework from qualitative study of healthcare managers (n =22) and professionals (n = 12) | MDCS-instrument (75 items)  *Providing resources and opportunities for digital competence sharing (11 items)*  *Creating methods and practices of digital competence sharing (11 items)*  *Managing healthcare professionals’ digital competence (11 items)*  *Implementing intergenerational learning (6 items)*  *Creating a friendly and safe digital organizational atmosphere (11 items)*  *Promoting digital competence sharing through leadership (25 items)* |
| Phase II Content validity testing | |
| January-March 2024  First expert panel CVI (n=8) | MDCS-instrument (44)  *Providing resources and opportunities for digital competence sharing (5 items)*  *Creating methods and practices of digital competence sharing (8 items)*  *Managing healthcare professionals’ digital competence (10 items)*  *Implementing intergenerational learning (5 items)*  *Creating a friendly and safe digital organizational atmosphere (9 items)*  *Promoting digital competence sharing through leadership (7 items)* |
| March 2024  Second expert panel, discussion (n=6) | MDCS-instrument (40 items)  *Providing* resources and opportunities for digital competence sharing (5 items)  *Creating methods and practices of digital competence sharing (8 items*  *Managing healthcare professionals’ digital competence (11 items)*  *Creating a friendly and safe digital organizational atmosphere (9 items)*  *Promoting digital competence sharing through leadership (7 items)* |
| Pretest | Minor changes to two background questions |
| Phase III Construct validity and reliability testing | |
| August 2024 -October 2024  Cross-sectional survey study of healthcare professionals (n=227) | MDCS-instrument (34 items)  *Creating a friendly and safe digital organizational atmosphere (8 items)*  *Creating methods and practices of digital competence sharing (8 items*  *Identifying and utilising professionals' digital competence (7 items)*  *Providing resources and opportunities for digital competence sharing (5 items)*  *Promoting digital competence sharing through leadership (6 items)* |
